# Supplementary material for: Exploring the Structure and Substance Metabolism of a Medicago sativa L. Stem Base
Source: Int J Mol Sci. 2024 Jun 5;25(11):6225. doi: 10.3390/ijms25116225 (PMC11172634; doi:10.3390/ijms25116225)
Supplement: Supplementary file 1 [file ijms-25-06225-s001.zip › ijms-2991357-supplementary/supplementary/accompanying drawing.pdf]

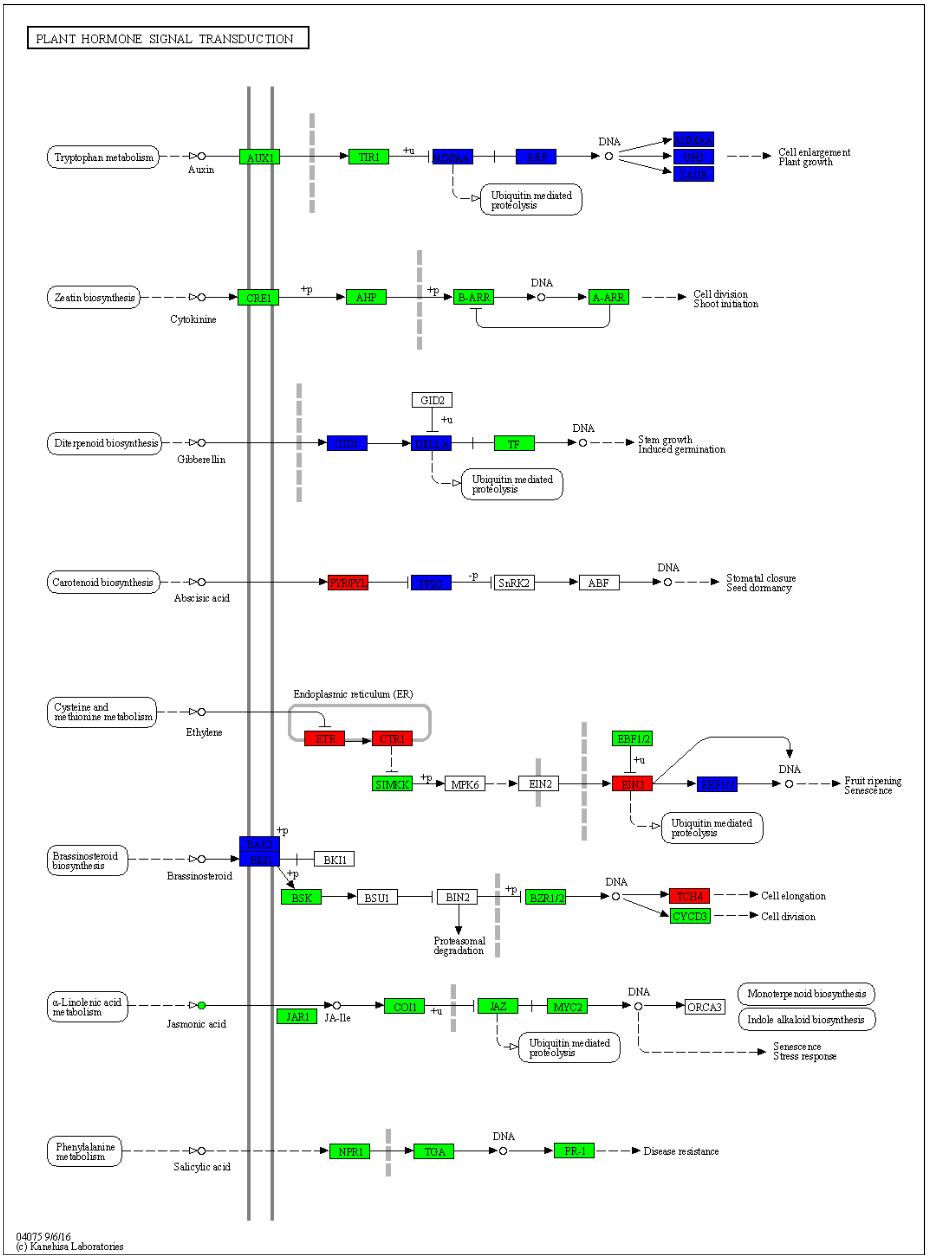

Figure S1. A diagram of the DACs and DEGs involved into plant hormone signal transduction(c-s).

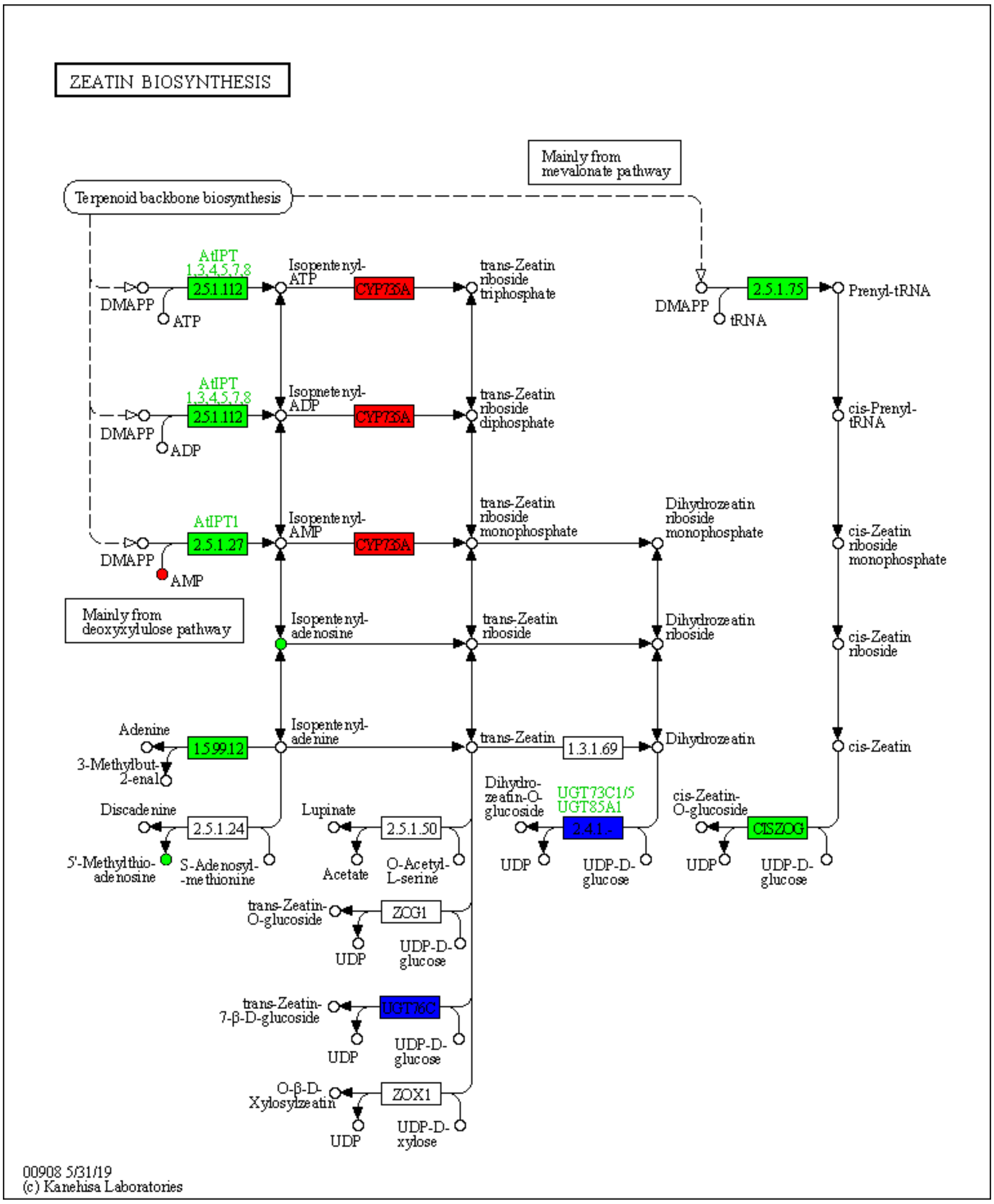

Figure S2. A diagram of the DACs and DEGs involved into zeatin biosynthesis(c-s).

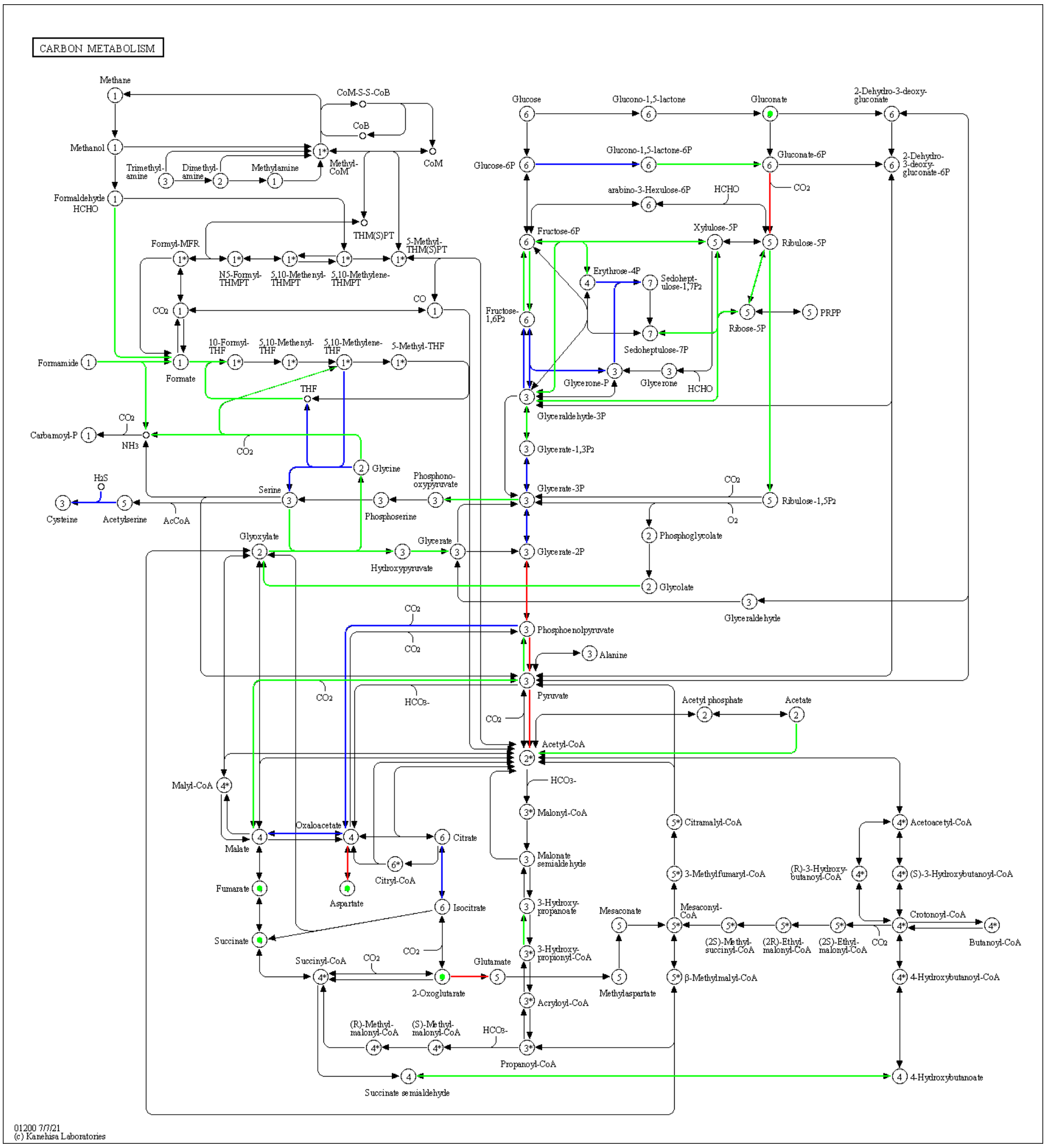

Figure S3. A diagram of the DACs and DEGs involved into carbon metabolism(c-s).

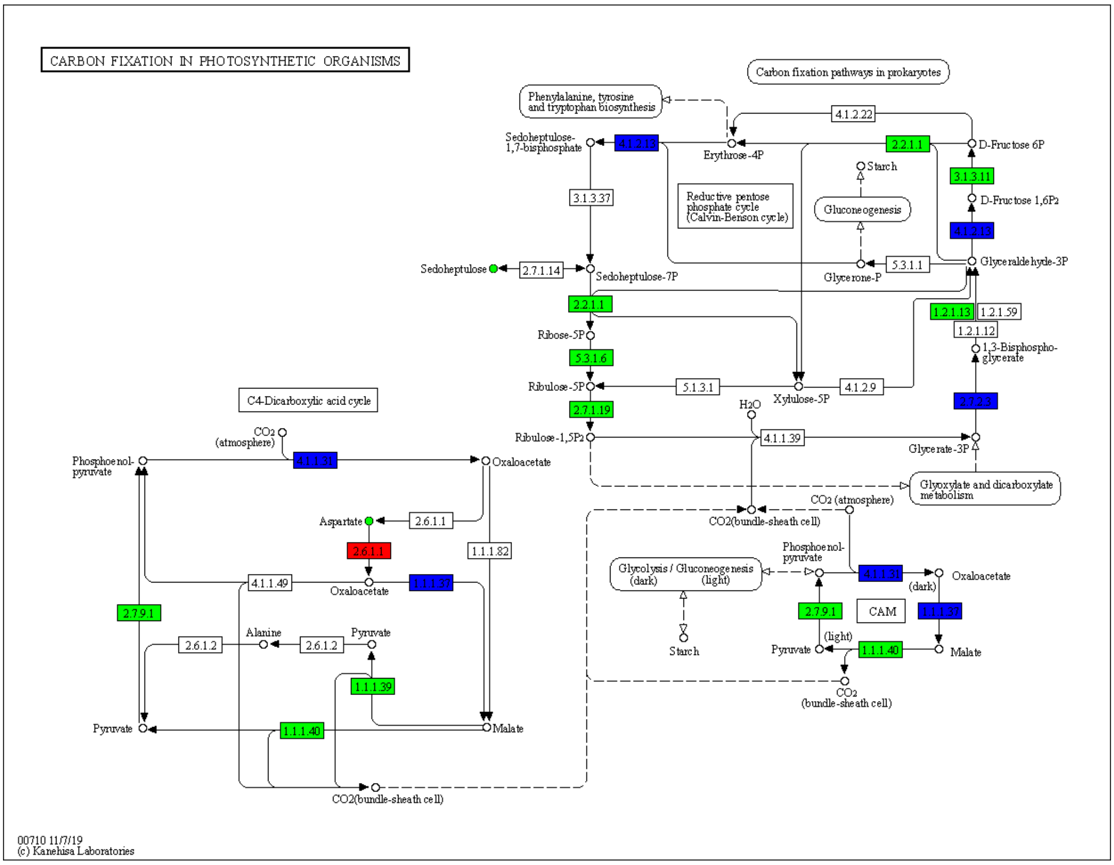

Figure S4. A diagram of the DACs and DEGs involved into carbon fixation in photosynthetic organisms(c-s).

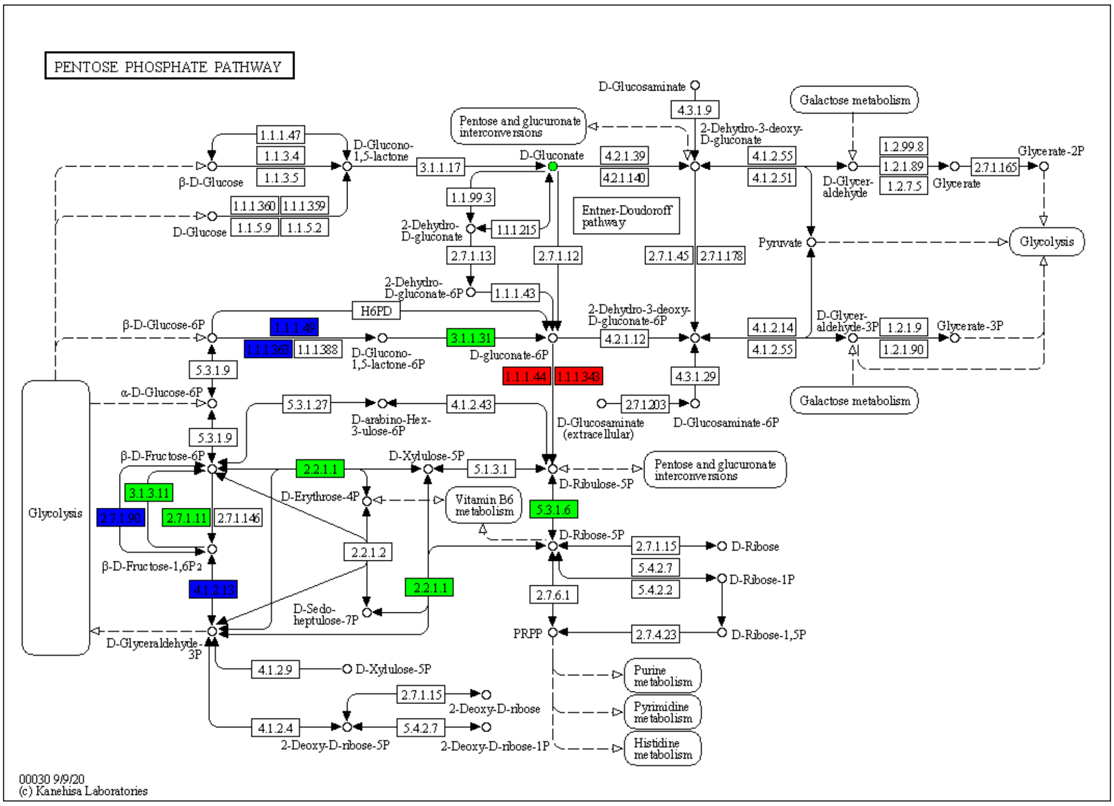

Figure S5. A diagram of the DACs and DEGs involved into pentose phosphate pathway(c-s).

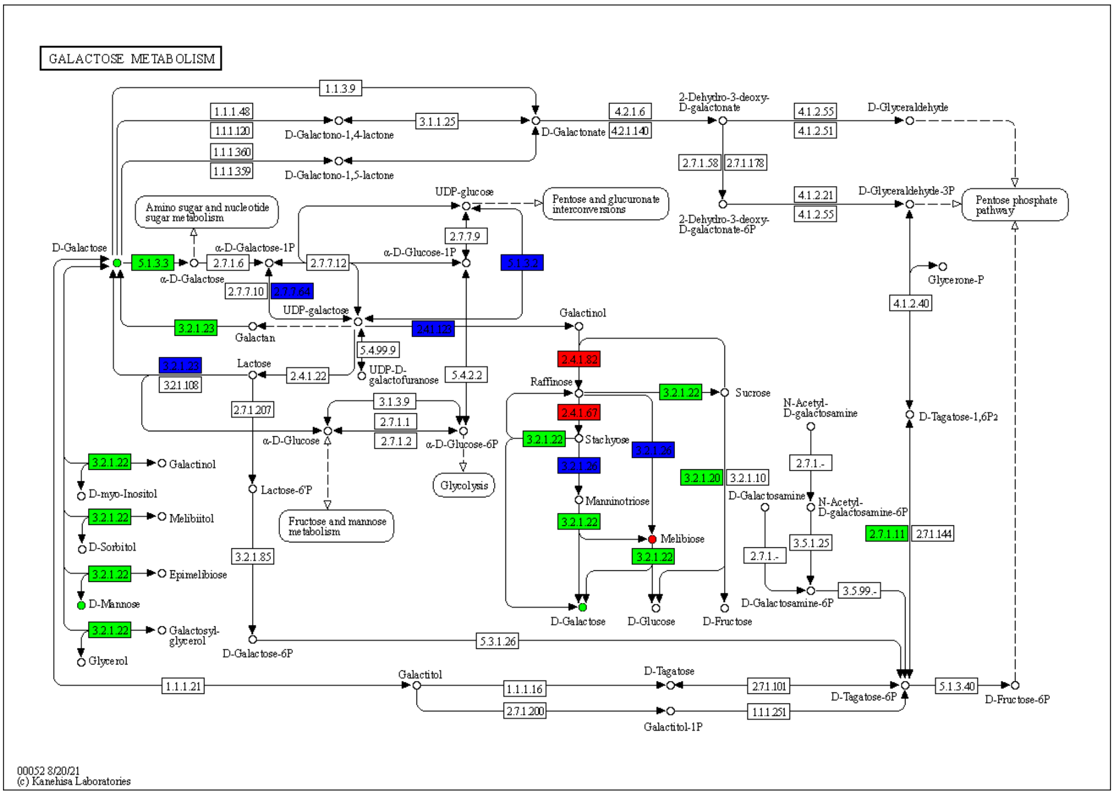

Figure S6. A diagram of the DACs and DEGs involved into galactose metabolism(c-s).

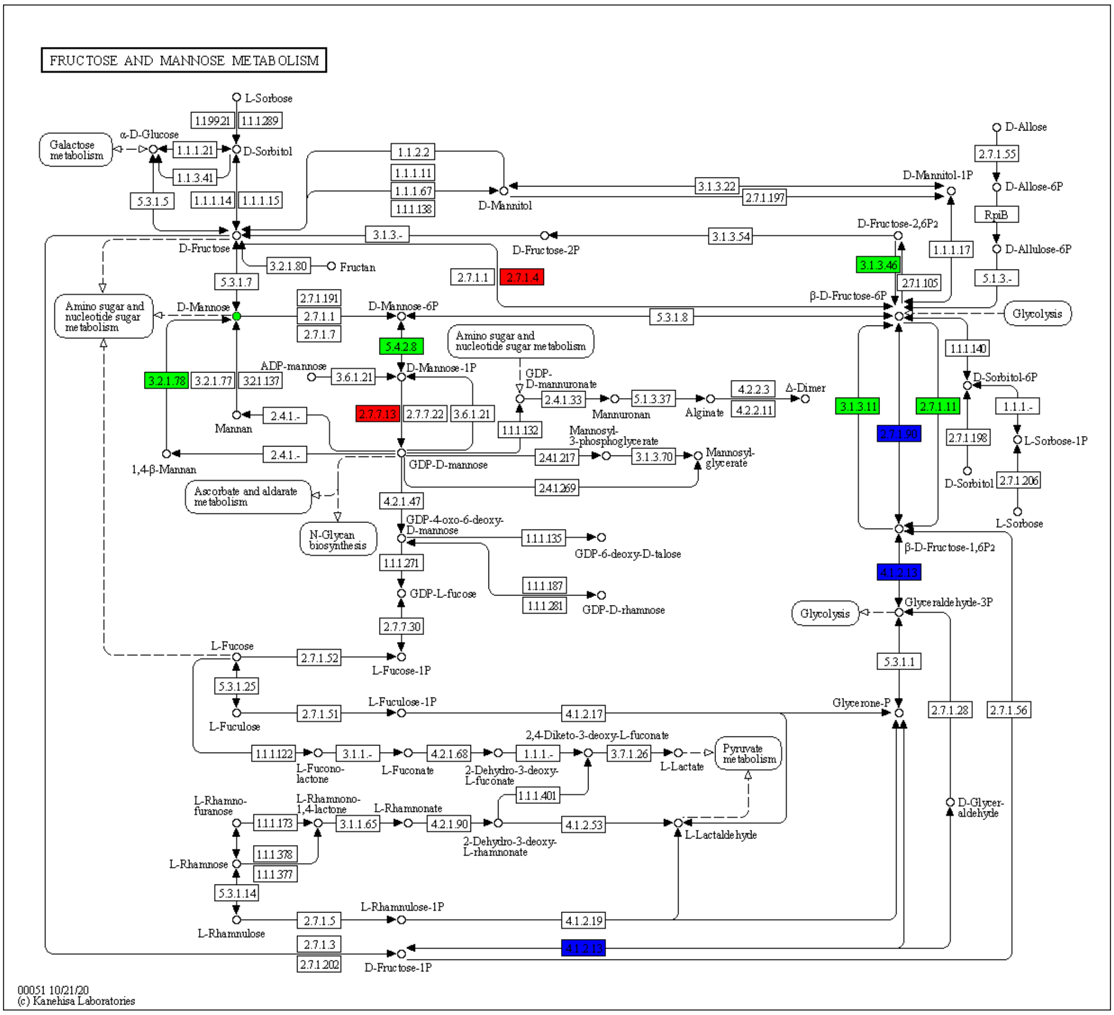

Figure S7. A diagram of the DACs and DEGs involved into fructose and mannose metabolism(c-s).

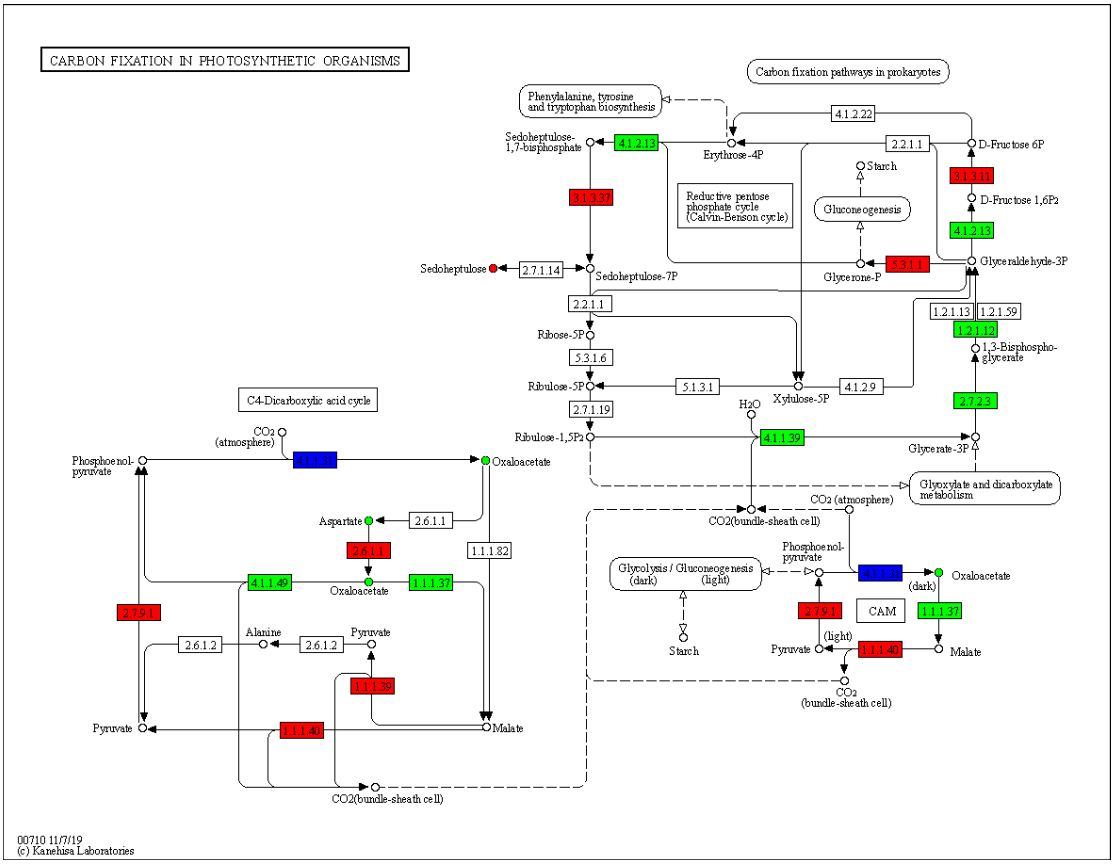

Figure S8. A diagram of the DACs and DEGs involved into carbon fixation in photosynthetic organisms(c-r).

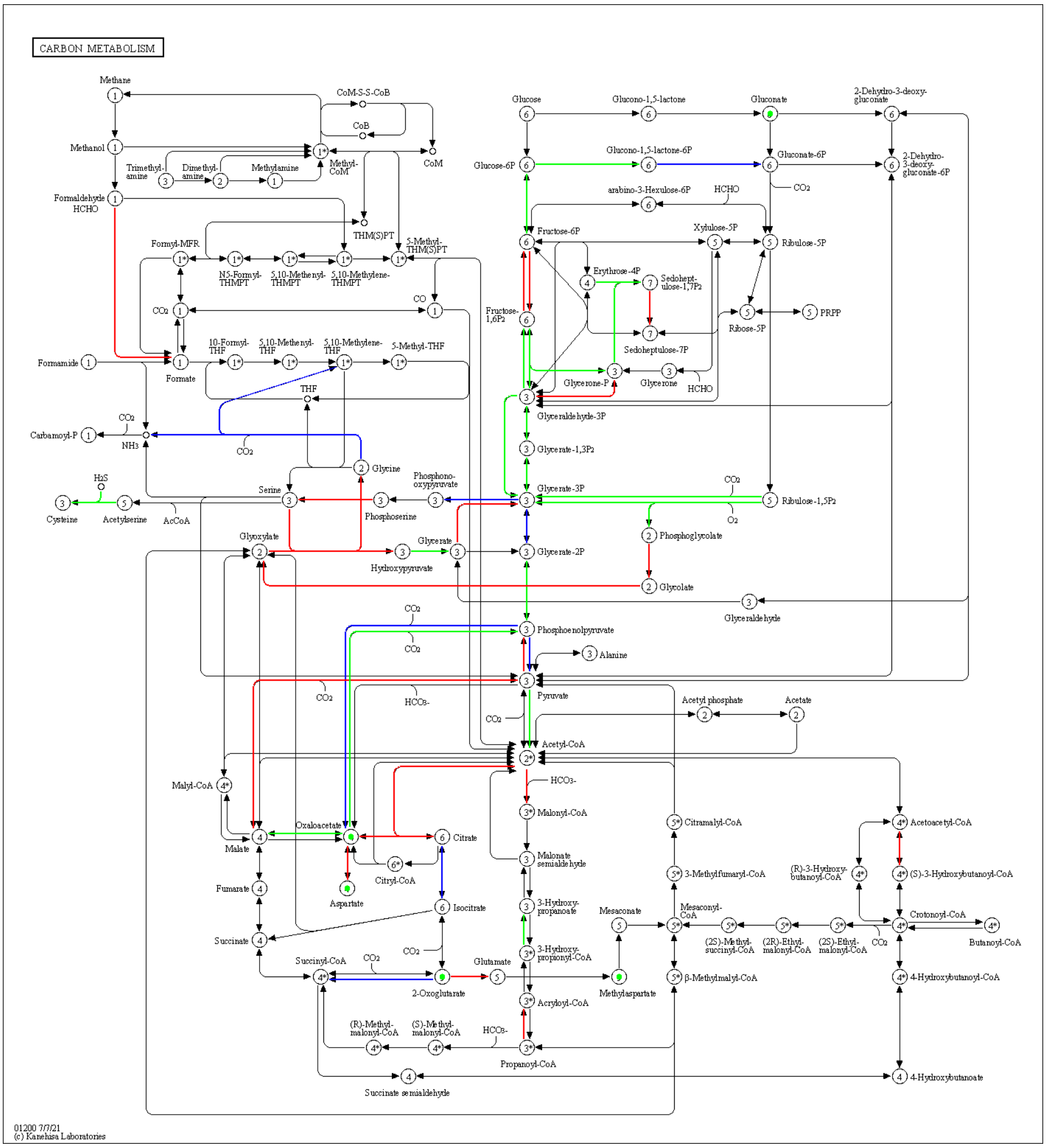

Figure S9. A diagram of the DACs and DEGs involved into fructose and mannose metabolism(c-r).

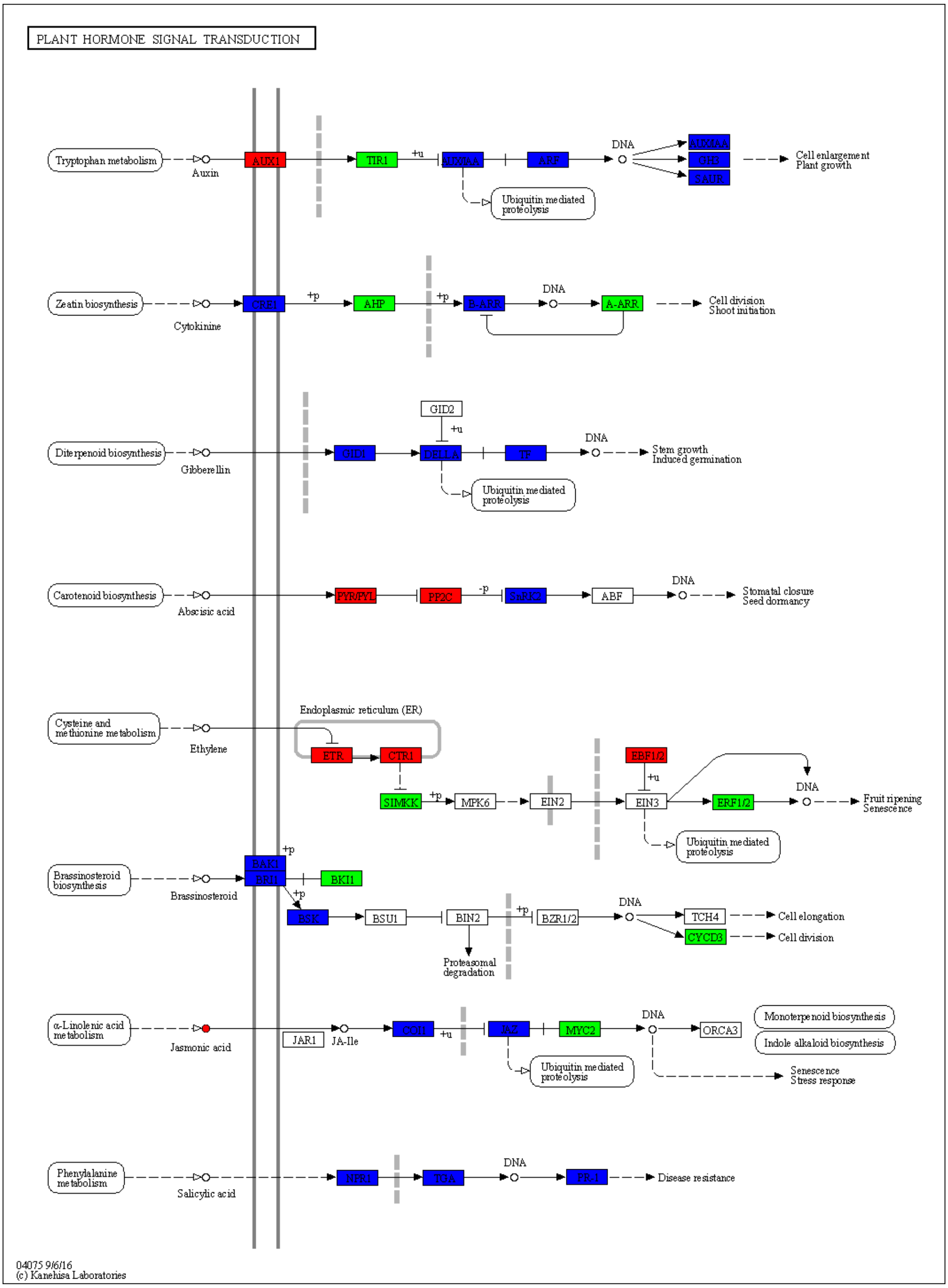

Figure S10. A diagram of the DACs and DEGs involved into fructose and mannose metabolism(c-r).

Note: The dots and boxes in the figure represent metabolites and genes, respectively. Red color indicates up-regulation of genes/ metabolites, green color indicates down-regulation of genes/ metabolites, and blue color indicates the presence of both up- and down-regulated genes.

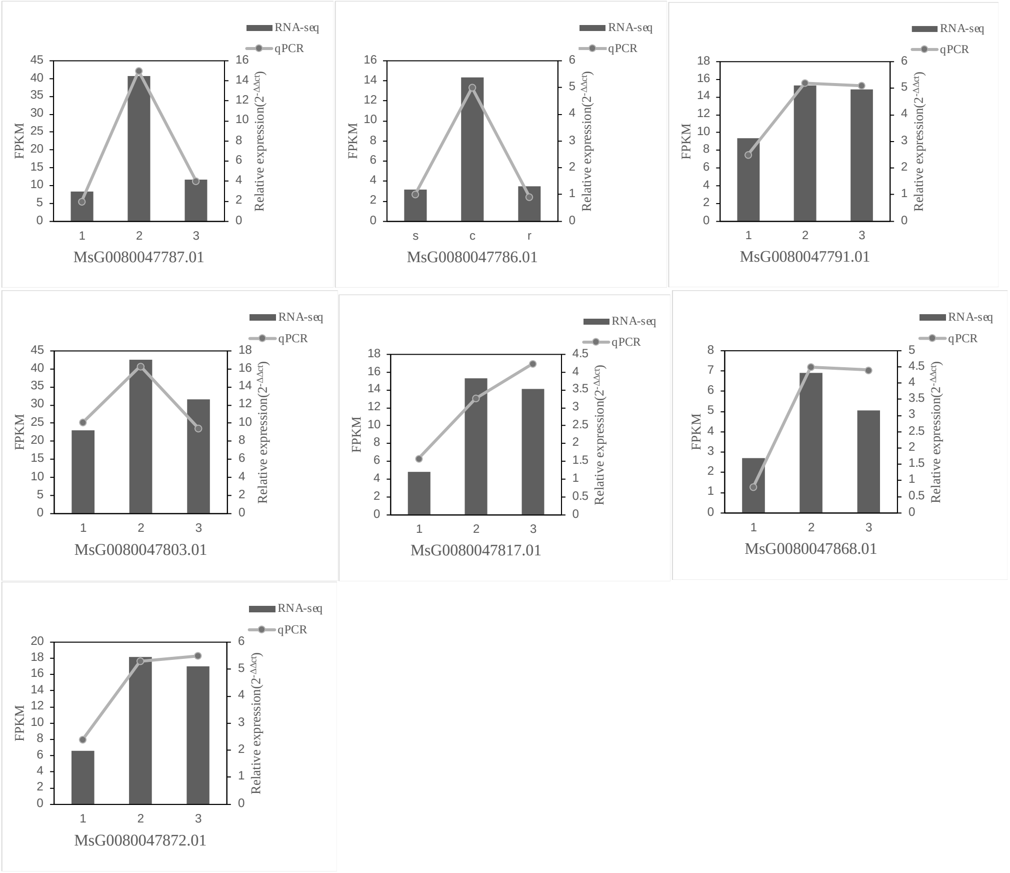

Figure S11. Verification of DEGs by qRT-PCR.

Note: Data are the means of three replicates.
